# Control of mRNA Translation in ALS Proteinopathy

**DOI:** 10.3389/fnmol.2017.00085

**Published:** 2017-03-23

**Authors:** Gianluca Cestra, Simona Rossi, Michela Di Salvio, Mauro Cozzolino

**Affiliations:** ^1^Institute of Biology and Molecular Pathology (IBPM), CNRRome, Italy; ^2^Department of Biology and Biotechnology Charles Darwin, University of Rome “Sapienza”Rome, Italy; ^3^Institute of Translational Pharmacology (IFT), CNRRome, Italy

**Keywords:** RNA translation, stress granules, eIF2α, amyotrophic lateral sclerosis (ALS), proteotoxic stress

## Abstract

Cells robustly reprogram gene expression during stress generated by protein misfolding and aggregation. In this condition, cells assemble the bulk of mRNAs into translationally silent stress granules (SGs), while they sustain the translation of specific mRNAs coding for proteins that are needed to overcome cellular stress. Alterations of this process are deeply associated to neurodegeneration. This is the case of amyotrophic lateral sclerosis (ALS), a neurodegenerative disorder caused by a selective loss of motor neurons. Indeed, impairment of protein homeostasis as well as alterations of RNA metabolism are now recognized as major players in the pathogenesis of ALS. In particular, evidence shows that defective mRNA transport and translation are implicated. Here, we provide a review of what is currently known about altered mRNA translation in ALS and how this impacts on the ability of affected cells to cope with proteotoxic stress.

## Introduction

Amyotrophic lateral sclerosis (ALS) is a progressive, adult-onset neurodegenerative disease. The most significant feature of ALS is the preferential loss of motor neurons from brain and spinal cord. Yet, a huge heterogeneity characterizes ALS manifestations, and it is now accepted that ALS is a complex multi-systemic syndrome with important aspects that overlap with other neurodegenerative disorders (Swinnen and Robberecht, 2014). A wealth of mechanisms has been suggested to cause ALS pathogenesis, such as protein aggregation, oxidative stress, mitochondrial damage, excitotoxicity and RNA dys-metabolism (Taylor et al., 2016). Although a big effort is still needed to clarify whether and to which extent each of these mechanisms play a role, key aspects of disease pathogenesis have clearly emerged.

The most relevant ALS genes give rise to proteins that accumulate as misfolded and/or aggregated species inside cells, both as wild-type or mutated forms. Indeed, protein aggregates are readily found in SOD1-, TDP-43-, FUS- and C9orf72-ALS, the most frequent genetic forms of the disease. Accordingly, impaired protein homeostasis, a condition that is almost invariably associated to misfolding and aggregation, has been frequently reported in ALS (Ruegsegger and Saxena, 2016). Moreover, an early and specific induction of endoplasmic reticulum (ER) stress has been proposed to explain the selective vulnerability of motor neurons (Ng et al., 2015; Sun et al., 2015), suggesting that these alterations might play a key role in disease pathogenesis. This conclusion is further supported by the evidence that a number of genes that participate to the autophagic and proteasomal control of protein degradation, such as ubiquilin-2 (Deng et al., 2011), sequestosome-1 (Fecto et al., 2011), optineurin (Maruyama et al., 2010), valosin-containing protein (VCP; Johnson et al., 2010), TBK1 (Freischmidt et al., 2015) and VAPB (Chen et al., 2010), are associated to ALS.

FUS and TDP-43, as well as other ALS-related genes, have also a recognized role in the regulation of RNA metabolism, including RNA transcription and splicing, microRNA processing and mRNA stability, transport and translation (thoroughly reviewed in Ratti and Buratti, 2016). Additionally, the expanded G4C2 hexanucleotide repeat in the C9orf72 gene, the most frequent genetic mutation in ALS, might impact the proper regulation of RNA metabolism, as a result of both the accumulation of RNAs containing the expansion, that might disrupt the activity of a number of RNA binding proteins (RBPs), and the expansion-driven production of poly-dipeptides (dipeptide repeat proteins, DPRs), that accumulate in cells and affect the proper intracellular trafficking of RNAs (Wen et al., 2017). Although a specific step of RNA processing that is predominantly affected in motor neurons has not emerged so far, mRNA translation might represent a central process in ALS, as alterations in the control of translation might directly affect motor neuron functions and their ability to regulate translation in condition of proteotoxic stress.

## Alterations of Translational Control in ALS

Protein synthesis is defined by the three classical phases that include initiation, elongation and termination (Aitken and Lorsch, 2012; Hinnebusch et al., 2016). Translation initiation is the rate-limiting step and the most tightly regulated stage, in which the two ribosomal subunits are assembled into 80S ribosomes with methionyl-tRNA paired to the mRNA start codon. In the canonical, cap-dependent translation initiation, once the GTP-bound form of the eukaryotic initiation factor eIF2 is loaded with the methionyl-tRNA, it builds the ternary complex. This binds the small ribosome subunit and generates the 43S preinitiation complex. Such 43S complex is assembled on the 5′ mRNA methyl cap by the initiation factor eIF3, with the contribution of the polyA-binding protein and the eIF4F complex. This is a crucial step, as mechanisms that inhibit eIF4F assembly will in turn inhibit translation initiation. Once formed, the 43S preinitiation complex walks along the mRNA searching for an initiation AUG codon positioned in the optimal sequence context that is necessary for the establishing of an effective 48S preinitiation complex. Arrest of the scanning process is caused by the codon-anticodon recognition, which elicits eIF5B GAP activity and thereby promoting the hydrolysis of the GTP in the ternary complex. eIF2-GDP is detached from the ribosome and this eventually leads to the formation of the 80S ribosomal complex. Subsequently, translation elongation factors (eEF) are recruited to promote polypeptide chain extension until the cycle is ended by the action of stop codon-dependent termination factors. In this process, the alpha subunit of eIF2 has a critical role. Indeed, phosphorylation of eIF2α by stress-induced kinases is the leading mechanism that inhibits the formation of functional translation initiation complexes and causes mRNA accumulation into stress granules (SGs; Spriggs et al., 2010), leading to translational repression of the bulk of mRNAs in favor of those mRNAs that are required to subside stress. Recent studies support an intimate relationship between ALS pathogenesis and protein synthesis: several ALS causative mutations affect the process of mRNA translation; novel unconventional and still unveiled translational processes, such as repeat associated non-AUG (RAN) translation, contribute to the pathogenesis of ALS.

### Defects in mRNA Transport and Local Translation

Modification of neuronal activity ultimately depends on the precise localization and turnover of synaptic proteins such as neurotransmitters, channels, adapters and signaling molecules. Synaptic accumulation of these proteins is also achieved by local translation of specific mRNAs peripherally transported by RNA granules (Krichevsky and Kosik, 2001): ribonucleoprotein complexes containing ribosomes and temporary translationally arrested mRNAs, which are locally released and translated in response to appropriate synaptic stimuli. Several RBPs play a pivotal role in the assembly and control of RNA transport granules, by assisting mRNAs in their way from the cell body to the periphery and modulating their stability and translation.

Evidence suggests a direct association of dysfunctional neuronal transport granules with ALS. Both TDP-43 and FUS are found in RNA transport granules (Kanai et al., 2004; Elvira et al., 2006), and ALS-linked mutant TDP-43 affect their trafficking (Alami et al., 2014). FMRP, a translational repressor causally linked to Fragile X Syndrome, also associates to RNA transport granules, and interacts with TDP-43. FMRP expression in *Drosophila* robustly modifies TDP-43-mediated neurodegeneration. Further, both FMRP and TDP-43 collaborate to repress the translation initiation of mRNAs encoding proteins that are essential for synapse formation and plasticity such as Rac1, GluR1 and Map1b (Majumder et al., 2012, 2016; Coyne et al., 2014, 2015; Romano et al., 2016). Although the localization of FUS in motor neuron synaptic terminals has not been unambiguously clarified, recent findings suggest an involvement of FUS in the regulation of local translation. In non-neuronal cells FUS binds to the tumor-suppressor protein adenomatous polyposis coli (APC), which forms ribonucleoprotein complexes for targeting mRNAs to cell protrusions, and is required for efficient local translation of transcripts associated to peripheral cellular processes (Yasuda et al., 2013). In our previous work, we demonstrated that FUS carrying ALS causative mutations physically and genetically interacts with Pur-alpha (Di Salvio et al., 2015), a DNA-RNA binding protein with several functions, including the targeting of mRNAs to neuronal dendrites, as part of RNA transport granules. In particular, Pur-alpha behaves as a regulator of SG assembly and, together with FUS, inhibits protein synthesis, participating to FUS toxicity (Di Salvio et al., 2015). Intriguingly, Pur-alpha also binds to G4C2 expanded repeats of C9orf72 gene and ameliorates G4C2-mediated neurodegeneration in *Drosophila* (Xu et al., 2013).

### RAN Dipeptides and mRNA Translation

Secondary and tertiary structures in the 5′ UTR of mRNAs strongly affect translation initiation efficiency (Green et al., 2016). When these regions are placed downstream the start codon, they intensely increase translation initiation, either in the case of suboptimal AUG or even in the total absence of a start codon (Green et al., 2016). This is particularly relevant in RAN translation, where translation starts in the absence of start codon and proceeds at multiple reading frames generating repeat-containing proteins. Both strands of C9orf72 gene carrying expanded repeats are actively transcribed, and both corresponding RNAs are expected to form stable secondary structures. Accordingly, both transcripts generate different DPRs by RAN translation: GA, GR, PA, PR and GP. The molecular machinery that controls RAN translation still needs to be fully defined (Green et al., 2016). However, recent key findings indicate that it uses a conventional cap-dependent ribosomal scanning, but bypasses normal requirements for start codon selection (Kearse et al., 2016). The formation of DPRs is widely believed to play a relevant role in C9orf72 toxicity. In particular, DPRs have been associated with functional alterations of proteins belonging to RNA transport granules. By analyzing the DPR interactome, it has clearly emerged, in fact, that the more toxic dipeptides GR and PR specifically bind to RBPs of the axonal RNA transport granules and to proteins of the translational machinery (Tao et al., 2015; Kanekura et al., 2016; Lee et al., 2016). In particular, GR and PR affect two translation related pathways: they localize in the nucleolus, where they block ribosomal RNA synthesis, and they alter SG formation in the cytoplasm. Further, PR makes complexes with mRNAs and prevents the access of translation factors (Kanekura et al., 2016), evidence that underlines, once more, the strong association between alterations in protein synthesis and the pathogenesis of ALS.

## Regulation of Translation in Condition of Proteotoxic Stress

### Stress Granules in ALS

Several of the RBPs connected to ALS localize into SGs. These are membrane-less, hydrogel-like structures that store translationally arrested mRNAs that accumulate during cellular stress, and that rapidly disassemble when stress is removed, in a process generally referred as “SG dynamics”. SGs are composed by polyadenylated mRNAs, translation initiation factors (eIFs), small ribosomal subunits and a great number of RBPs (Protter and Parker, 2016), including ALS-linked RBPs. Among the latter, FUS and TDP-43 represent the most notable example of proteins that shuttle from cell nuclei, where they predominantly reside, into cytosolic SGs in response to a vast array of stress stimuli. This ability is held by wild type forms of both TDP-43 and FUS, that readily localize into SGs in stress conditions, and by ALS-linked mutant forms, that show an enhanced propensity to coalesce into SGs (Bosco et al., 2010).

In addition to TDP-43 and FUS, also hnRNP A2/B1 and hnRNP A1 (heterogeneous nuclear ribonucleoprotein A2/B1 and A1), two RBPs whose mutations are associated to rare forms of familial ALS (Kim et al., 2013), are part of SGs under stress conditions. This suggests a role of these two hnRNPs in stress response that might be impaired, by still unknown mechanisms, by their mutations (Guil et al., 2006; Martinez et al., 2016). Further, Ataxin 2, an ALS-linked RBP that causes spinocerebellar ataxia type 2 (SCA2), is associated to SGs and might have a role in their assembly (Nonhoff et al., 2007; Kaehler et al., 2012). However, also non RBP-like ALS factors, that are not obviously expected to be part of SGs, are indeed found into these structures. Profilin1, an actin binding protein whose mutations are a rare cause of familial ALS, localizes into SGs upon induction (Figley et al., 2014). Furthermore, mutant forms of SOD1, which are associated to frequent forms of familial ALS, may relocalize to SGs. Mutant SOD1s bind G3BP1, an RBP that plays important roles in SGs dynamics, and affect its ability to bind RNAs, thus interfering with SG assembly. As a result, the formation of SGs in response to stress is delayed or impaired, with obvious consequences on cell viability (Gal et al., 2016). Notably, a similar scenario seems to operate in C9orf72-ALS, where GR and PR dipeptides promote the spontaneous assembly of SGs and a consequent inhibition of global protein translation (Wen et al., 2014; Kanekura et al., 2016; Lee et al., 2016). Both GR and PR interact with G3BP1, suggesting that the simple interaction with G3BP1 is sufficient to impair SG dynamics, as in the case of SOD1 mutations, and that the accumulation of DPRs into SGs could be not required to alter their function (Lee et al., 2016).

Therefore, SGs might strongly contribute to ALS pathogenesis. Yet, how this occurs is still unclear. The tendency of ALS-related proteins to accumulate into SGs might play a relevant role in the formation of cytosolic aggregates that mark the affected tissues in ALS patients. Indeed, the intrinsic propensity of many RBPs to reversibly associate to each other (King et al., 2012) might act as a seed for the nucleation of intracellular aggregates when prolonged stress and/or mutations occur (Li et al., 2013). Nevertheless, evidence also exists that aggregates might originate independently (Shelkovnikova et al., 2014).

However, the association of ALS proteins to SGs supports a model whereby the disease might be the consequence of a disturbed SG regulation in both unstressed conditions, when mutant ALS factors might induce an uncontrolled and sustained activation of SG-mediated translational repression, or under stress, when such a response, that is usually strictly and finely tuned, might escape this regulation. Three lines of evidence support this model. First, genes that are involved in SG regulation are potent modifiers of the toxicity of mutant ALS genes, including TDP-43, Profilin 1 and C9orf72, in yeast and *Drosophila* (Couthouis et al., 2011; Kim et al., 2014; Lee et al., 2016). Second, several ALS-linked genes have a physiological role in SG formation and dynamics, and the loss of these functions might be involved in ALS (Aulas et al., 2012, 2015). Last, mutant ALS proteins might interfere with chaperone-mediated clearance from SGs of DRiPs, Defective Ribosomal Products constituted by terminated polypeptides that are released by disassembling polysomes prior to SG formation. Altered clearance of these peptides might lead to a decreased rate of SG disassembly, which further indicates that ALS can be associated with a protracted state of stress and the consequences of such a state (Ganassi et al., 2016).

### Role of eIF2α Phosphorylation in ALS Pathology

Being the key factor in the regulation of SG formation upon stress conditions, eIF2α has attracted a large interest as a potential target for interventions aimed at modulating the stress response that is induced in different neurodegenerative conditions where proteotoxic stress occurs. Following the activation of the Unfolded Protein Response (UPR) by high levels of unfolded or misfolded proteins, the PERK kinase phosphorylates eIF2α. This decreases the active pool of the ternary complex with the consequent inhibition of global translation. This pathway is indeed involved in ALS pathology, since ER stress markers, including phosphorylated eIF2α, have been detected in tissues from ALS patients and animal models (Matus et al., 2013). However, whether the sustained activation of this response represents a protective mechanism or has a pathological role is unclear. This issue is of crucial importance, as it might help in defining whether enhancing or suppressing eIF2α phosphorylation might be effective in contrasting the disease. To clarify this issue, the effects of modulation of eIF2α phosphorylation on neurodegeneration have been largely investigated in different disease models.

Genetic approaches have been used to modulate the eIF2α-P levels in mice overexpressing mutated G85R-SOD1. In particular, PERK haploinsufficiency accelerates SOD1 misfolding, causes an earlier disease onset and shortens survival (Wang et al., 2011). Opposite effects with a marked amelioration of disease were obtained by expressing in G85R-SOD1 mice an inactive form of GADD34, the eIF2α-P specific phosphatase (Wang et al., 2014a). In line with these results, pharmacological treatment of SOD1-G93A mice with Salubrinal, Guanabenz, or its derivative Sephin, all inhibitors of eIF2α de-phosphorylation, delays disease progression and prolongs survival (Saxena et al., 2009; Wang et al., 2014b; Das et al., 2015). Altogether, these data indicate that UPR has an important role in SOD1-mediated ALS, and support the idea that the induction of UPR and the prolonged activation of eIF2α phosphorylation are beneficial to motor neuron survival, which is in line with the notion that stress response is essential for cell survival. However, in *Drosophila* the knockdown of PERK or GADD34 fly homologs respectively suppresses or enhances motility defects caused by TDP-43 toxicity (Kim et al., 2014). Moreover, a significant rescue of TDP-43 toxicity is observed in *Drosophila* and in primary rat cortical neurons followed by treatment with a specific PERK inhibitor (GSK2606414; Kim et al., 2014). The inhibition of the UPR pathway is also beneficial in other misfolding-related diseases, such as Prion Disease, Frontotemporal Dementia and Alzheimer (Moreno et al., 2012, 2013; Ma et al., 2013; Devi and Ohno, 2014; Radford et al., 2015). Taken together, these observations converge on the idea that while a transient eIF2α phosphorylation is essential to overcome stress conditions, the persistent expression of eIF2α-P and the associated translational repression could be detrimental for neuronal viability and contribute to the pathogenic mechanisms. Moreover, they indicate that in ALS other pathways that converge on the regulation of protein translation might be activated together with, or even independently from misfolding-induced stress and might explain the different response to eIF2α-P targeting. In particular, this is suggested by the actual role that ALS factors such as TDP-43 and FUS might have in the control of protein translation, as previously mentioned, but also by the observation that stress response seems to be readily activated in C9orf72 ALS even in the absence of a clear phosphorylation of eIF2α. Indeed, accumulation of C9orf72 repeats causes a general impairment in nucleo-cytosol shuttling of RNAs (Freibaum et al., 2015; Rossi et al., 2015), leading to an entrapment in cell nuclei of polyadenylated mRNAs, which would be therefore less available for cytosolic translation. Interestingly, similar conclusions were drawn in cultured cells expressing arginine-containing polypeptides (PR and GR), that bind mRNAs and interfere with eIF4F assembly, thus inhibiting protein translation in the absence of enhanced eIF2α phosphorylation (Kanekura et al., 2016).

**Figure 1:**
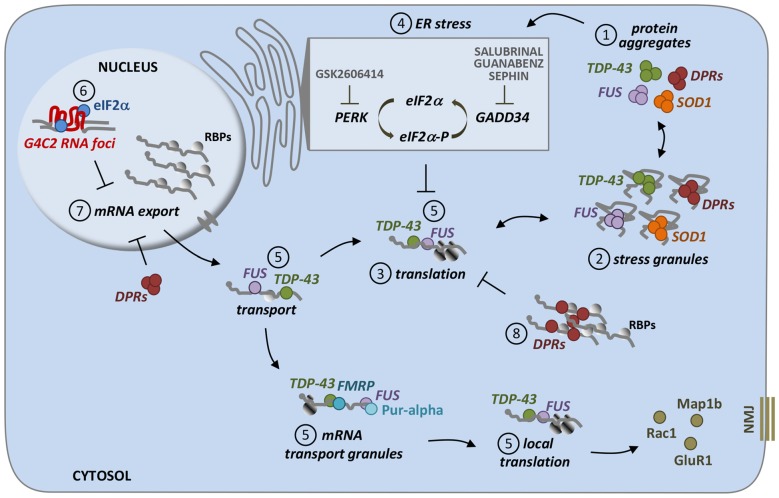
**Different pathways induced by ALS proteins converge on translational repression.** Most of ALS proteins, including mutant SOD1, TDP-43, FUS, as well as DPRs (poly-dipeptide repeat proteins which are RAN-translated from G4C2 repeat expansion in C9orf72 gene), accumulate as misfolded and/or aggregated species inside cells **(1)**. Interestingly, these protein aggregates are often found to be positive for stress granules markers suggesting a direct relationship between these two entities **(2)**. SG formation is strongly associated to translational repression, which is indeed observed in ALS models linked to these genetic forms **(3)**. Protein aggregates are able to induce ER stress, which is mediated by the activation of PERK kinase which in turn phosphorylates the translational initiation factor eIF2a (eIF2a-P; **4**). eIF2a-P has a central role in cell stress response, since it represses the translation of most cellular mRNA, while promoting the synthesis of proteins that help to overcome stress, including chaperones. Dephosphorylation of eIF2a, mediated by the phosphatase GADD34, is then essential to turn off this pathway. Drugs such as GSK2606414, Salubrinal, Guanabenz and Sephin, that act on this mechanism have been tested in models for their therapeutic efficacy, giving conflicting results. Other ways, which are independent from eIF2a phosphorylation, might be responsible of translational repression induced by ALS proteins. Indeed, FUS and TDP-43 are both RNA-binding proteins involved in RNA transport as part of RNA transport granules, and ALS-associated mutations might affect the proper localization and translation of specific mRNAs (such as Rac1, GluR1 and Map1b) that are important for neuromuscular junction (NMJ) structure and function **(5)**. Moreover, C9orf72 RNA foci sequester eIF2a, which might be less available for mRNA translation, thus resulting in translational inhibition and stress response **(6)**. Finally, both repeat RNA foci and DPRs cause mRNA export defects, thus leading to the retention of mRNAs in cell nuclei and their reduced cytoplasmic availability **(7)**. DPRs have been also found to directly bind mRNAs, making them less accessible to translation initiation factors, thus inhibiting protein translation **(8)**.

In conclusion, an increasing amount of genetic and mechanistic indications point to alterations in the proper control of mRNA translation as critical players in ALS pathogenesis (Figure 1). Clearly, further investigations are needed to better define whether and to which extent protein misfolding and aggregation are the actual trigger of these events, how mutations affect the ability of ALS-linked genes to regulate mRNA transport and local protein synthesis, and how the therapeutic potential of targeting mRNA translation regulation can be converted into an effective treatment in different ALS conditions.

## Author Contributions

GC and MC formulated the concept of the manuscript. GC, SR and MC wrote the manuscript. SR and MDS executed complete drawing of the figure. All authors reviewed the manuscript.

## Conflict of Interest Statement

The authors declare that the research was conducted in the absence of any commercial or financial relationships that could be construed as a potential conflict of interest.
